# Maintaining stable symptom control in inflammatory bowel disease: a retrospective analysis of adherence, medication switches and the risk of relapse

**DOI:** 10.1111/apt.12396

**Published:** 2013-07-08

**Authors:** A Robinson, M Hankins, G Wiseman, M Jones

**Affiliations:** * Salford Royal NHS Foundation TrustSalford, UK; † Faculty of Health Sciences, University of SouthamptonSouthampton, UK; ‡ Medical Affairs, Warner Chilcott UK LtdWeybridge, UK; § Health Informatics Research, Sciensus LtdBrighton, UK

## Abstract

**Background** Maintenance therapy with 5-aminosalicylic acid (5-ASA) is a key strategy for preventing relapse in many patients with inflammatory bowel disease (IBD). Factors which disrupt 5–ASA delivery, such as non-adherence and 5-ASA switches, may destabilise symptom control.

**Aim** To investigate the impact of non-adherence and medication switches on stable symptom control in UK patients with IBD.

**Methods** A retrospective cohort study was conducted using a UK dispensing database. Adherence was analysed in randomised matched samples for each of the six leading oral mesalazine formulations, measured by medication possession ratio (MPR); MPR ≥80% was classified as adherent. Relationships among adherence, switch and relapse were analysed over 18 months in patients receiving continuous mesalazine therapy throughout a 6–month baseline period (primary subgroup analysis). Relapses of active ulcerative colitis were identified using a doubling of MPR as a proxy.

**Results** Only 39% of patients in the matched samples (*n* = 1200) were classed as adherent. No significant differences in adherence were observed among mesalazine formulations. In the primary subgroup analysis (*n* = 568), non-adherent patients had a significantly greater risk of relapse than adherent patients (RR = 1.44, 95% CI = 1.08–1.94; *P* = 0.014). Among adherent patients (*n* = 276), those who switched had a 3.5-fold greater risk of relapse than those who did not switch (95% CI = 1.16–10.62; *P* = 0.008).

**Conclusions** Both non-adherence and mesalazine switches in adherent patients were associated with significant increases in the risk of relapse, suggesting that disruption of mesalazine maintenance therapy may destabilise symptom control. These findings provide evidence to advocate caution when considering mesalazine switches for stable patients.

## Introduction

Maintenance therapy is a key therapeutic strategy for many patients with inflammatory bowel disease (IBD). For patients with ulcerative colitis (UC), in particular, 5–aminosalicylic acid (5-ASA) represents a key first-line treatment option,^1^–^2^ with a well-established efficacy and tolerability profile.^3^ It is postulated that consistent delivery of 5–ASA throughout the maintenance phase may be central to maintaining stability. Factors that disrupt 5–ASA delivery may therefore be anticipated to increase the risk of relapse.

In particular, non-adherence to 5-ASA maintenance therapy is an established and prevalent problem in IBD patients.^4^–^5^ Patients who are non-adherent do not receive a consistent and continuous supply of 5-ASA, and may therefore be expected to be at a greater risk of relapse than adherent patients. This hypothesis is borne out by research conducted in the US, as studies by Kane *et al*. and Khan *et al*. demonstrated that non-adherent patients have a significantly greater risk of relapse than adherent patients.^6^–^7^ However, there is a paucity of robust evidence on the impact of non-adherence to 5-ASA therapy in the UK setting.

Furthermore, the potential impact of switches between 5-ASA formulations on disease control represents an increasingly important consideration. One of the key current debates regarding 5-ASA concerns whether 5–ASA formulations should be considered non-interchangeable due to their unique release profiles.^8^–^9^ If this were the case, a switch between 5-ASA formulations would alter the delivery of 5-ASA to the gut, and this disruption could potentially have knock-on effects on disease control. However, to date, there is little direct evidence on the effect of 5-ASA switches on patients.

This study therefore aimed to investigate the prevalence and impact of factors that may destabilise symptom control in UK patients with IBD, using observational data from a drug dispensing database. The analysis focused first on adherence, controlling for confounding factors to obtain a standardised view. By establishing a practical proxy measure for clinical outcomes, the study then progressed to investigate the effect of 5-ASA switches on stable symptom control.

## Materials and methods

### Study design and patients

This was a retrospective cohort study using dispensed drug records from the CegedimRx Data Repository. This large UK dispensing database covers an estimated 7 million individuals and 1590 pharmacies across the UK, with a broad geographical coverage across the country. Adults who received at least two dispensed prescriptions for oral mesalazine between January 2008 and December 2011 were eligible for inclusion in the full data set. Patients were treatment naïve for all study drugs in the 6 months prior to the index date, to ensure a homogeneous population and avoid confounding due to patients' experiences (e.g. switches and relapses) prior to baseline.

**Figure 1:**
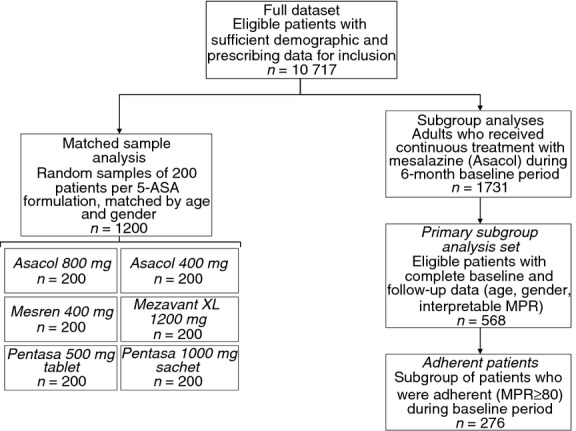
Patient disposition and analysis sets.

Analyses of adherence, switches and relapses of active UC were conducted in defined analysis sets drawn from the full data set (Figure 1). Evaluation of adherence was conducted in matched samples, to obtain a standardised view and minimise confounding. Each sample was selected randomly from eligible records for each of the leading formulations of oral mesalazine, matched by age and sex. The six mesalazine formulations included in this analysis were Asacol 400 mg and Asacol 800 mg MR oral tablets (Warner Chilcott UK Ltd, Weybridge, UK), Pentasa 500 mg slow release tablets and 1000 mg sachets (Ferring Pharmaceuticals Ltd, West Drayton, UK), Mesren MR 400 mg tablets (Teva Pharmaceuticals Ltd, Harlow, UK), and Mezavant XL 1200 mg gastro-resistant, prolonged release tablets (Shire Pharmaceuticals Ltd, Basingstoke, UK). Adherence was measured for the duration of continuous mesalazine therapy, over more than 3 years.

Analyses of mesalazine switches and relapses were conducted in defined subgroups of the full data set (Figure 1), followed over a 6-month baseline period and an 18-month follow-up period. To avoid potential confounding by mesalazine formulation, these analyses were restricted to a single formulation of oral mesalazine; to ensure sufficient statistical power, the most commonly prescribed mesalazine formulation was used. Thus, patients in the full data set who received continuous treatment with Asacol (400 mg MR or 800 mg MR oral tablets) during the baseline period were eligible for inclusion in the subgroup analyses. Patients for whom complete baseline and follow-up data were available were included in the primary subgroup analysis set. Relationships among adherence, switch and relapse were examined using cohort and nested case–control methods, controlled for age and sex.

### Study evaluations and measures

Adherence was measured using the medication possession ratio (MPR), calculated as the ratio of the intended prescription duration to the actual interval between prescription refills:


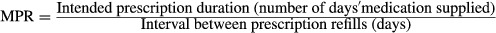


Medication possession ratio is one of the most widely used measures of adherence,^10^ is the accepted standard for measuring adherence in analyses such as this and has been shown to be significantly associated with clinical measures of adherence (e.g. serum drug levels, clinical drug effects).^11^ Patients with an average MPR ≥80% were classed as adherent; this is in line with previous adherence studies conducted in IBD.^6^–^12^ Patients with an average MPR >200% over the whole study period were excluded from the analysis. Ostensibly, excessive adherence rates typically arise through anomalies in dispensing or refill practice, for example, if a patient receives half of their prescribed medication in one dispensing followed by the remainder the following day, they would record an MPR of several thousand per cent. Such events are not clinically relevant, but may bias the overall adherence scores, so are excluded from this study.

Switches between mesalazine formulations were identified on the basis of a change to an alternative mesalazine formulation between successive prescription refills.

Dispensing databases do not include direct reporting of clinical events and outcomes. Consequently, relationships among adherence, mesalazine switches and clinical outcomes were assessed by identifying a proxy measure for relapse. A common clinical approach in many cases of relapse of active UC is to double the dosage of mesalazine: such an approach is broadly consistent with treatment guidelines from the British Society of Gastroenterology (BSG), which recommends mesalazine 1.2–2.4 g/day for maintenance therapy and 2.4–4.8 g/day for treatment of active disease.^2^ Events such as this are directly recorded in dispensing data, so this was identified as a possible proxy measure for relapses of active UC. Consequently, a ≥ 2-fold increase in MPR (indicating a doubling of mesalazine dose; termed peak MPR) was used as a proxy measure for relapse of active UC – i.e. the definition of relapse in this study was a ≥ 2-fold increase in MPR.

As the peak MPR measure was based on the mesalazine formulation being taken at the time (i.e. it was not compared to MPR before a switch), switches between formulations that were simultaneously accompanied by a doubling of dose were not recorded as a relapse in the follow-up period. Consequently, this proxy measure specifically identified relapses that occurred after a switch, and not concurrently; the results are not influenced by clinicians electing to treat a relapse by switching mesalazine treatment to another formulation at a doubled dose.

### Statistical analysis

In the matched sample analysis, comparisons of adherence among samples were conducted using logistic regression with adherence as a dichotomous outcome. For this analysis, assuming a baseline adherence of 40%, the sample size of 200 patients per group provided 80% power to detect a risk ratio of 1.50 or greater at the 5% significance level (two-tailed) after adjustment for multiple pairwise comparisons. In the subgroup analyses, comparisons of the risk of relapse among groups were conducted using chi-squared tests, adjusted for age and sex as required. Risk ratios were computed to express effect sizes. All statistical analyses were conducted using spss software (IBM).

## Results

### Demographic characteristics

A total of 10 717 records were extracted and provided sufficient demographic and prescribing data for inclusion in the full data set. Matched samples were identified for each of the six most commonly prescribed mesalazine formulations (Asacol 400 mg, Asacol 800 mg, Pentasa 500 mg tablets, Pentasa 1000 mg sachets, Mesren 400 mg, Mezavant XL 1200 mg), as described. Each sample comprised 200 patients, of whom 50% were male and the mean age was 50 years (Table 1). A total of 1731 patients were eligible for inclusion in the subgroup analysis, of whom 568 provided complete baseline and follow-up data for inclusion in the primary subgroup analysis set (51.2% male, mean age 56 years; Table 1).

**Table 1:** Baseline and demographic characteristics

	Matched samples	Primary subgroup analysis set	Adherent patients
*N*	1200	568	276
Age, years			
Mean	50	56.0	58.2
Range	18–99	18–91	20–91
Sex,% male	50%	51.2%	54.7%

### Adherence – matched sample analysis

**Figure 2:**
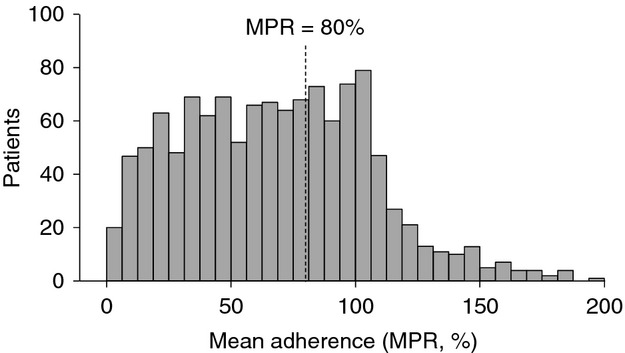
Distribution of adherence scores for patients treated with mesalazine in the matched sample analysis (*n* = 1200). MPR, medication possession ratio; patients with an MPR ≥80% were classed as adherent.

**Figure 3:**
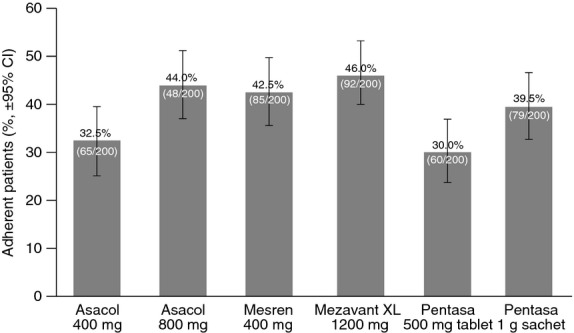
Percentage of patients classed as adherent (mean MPR ≥80%) in the matched sample analysis, by mesalazine formulation (*n* = 200 per formulation). MPR, medication possession ratio.

Overall, 469/1200 patients (39.1%) were classed as adherent (mean MPR ≥80%; Figure 2). No significant differences in adherence were observed among mesalazine formulations (*P* > 0.05, adjusted for multiple comparisons; Figure 3). Similar adherence scores were observed across the six leading mesalazine formulations, despite differences in the dosages and regimens for which they are indicated.

### Impact of non-adherence on relapse

**Figure 4:**
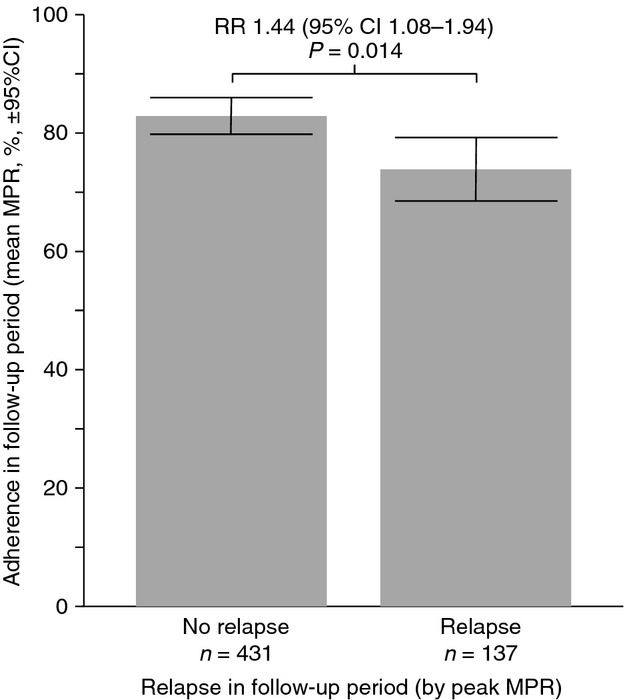
Relationship between adherence and relapse in a nested case–control analysis (primary subgroup analysis set, *n* = 568). MPR, medication possession ratio; RR, risk ratio.

The impact of non-adherence on relapse during the 18-month follow-up period was evaluated in the primary subgroup analysis set (*n* = 568). The mean duration of follow-up in this analysis was 600.29 days (1.64 years; 95% CI 572.2–628.4 days). Relapses were recorded in a total of 137/568 patients (24.1%) during the follow-up period. In a nested case–control analysis, the mean MPR in patients who experienced relapses in the follow-up period was 76.6%, compared to 82.8% in those who did not experience relapses (Figure 4). There was a significant association between non-adherence and relapse in the follow-up period, indicating that there was a significantly greater risk of relapse in patients who were non-adherent (MPR <80%; RR 1.44, 95% CI 1.08–1.94; *P* = 0.014).

These findings were corroborated by a corresponding analysis in the baseline period. Relapses were recorded in 15.8% of patients during the baseline period, and the mean adherence score for patients who experienced relapses was 66.1%, compared to 79.4% in those who did not experience relapses.

### Destabilisation of symptom control – effect of switches

In total, 501/568 patients (88.2%) switched mesalazine formulation during the study. In an initial examination of the full subgroup, there was no significant relationship between switch and either adherence or relapse. The mean MPR in the follow-up period for patients who switched mesalazine was 81.2%, compared to 78.6% in those who did not switch (*P* = 0.744). In the follow-up period, 24.4% of those who switched experienced a relapse, compared to 23.1% of those who did not switch (*P* = 0.820).

**Figure 5:**
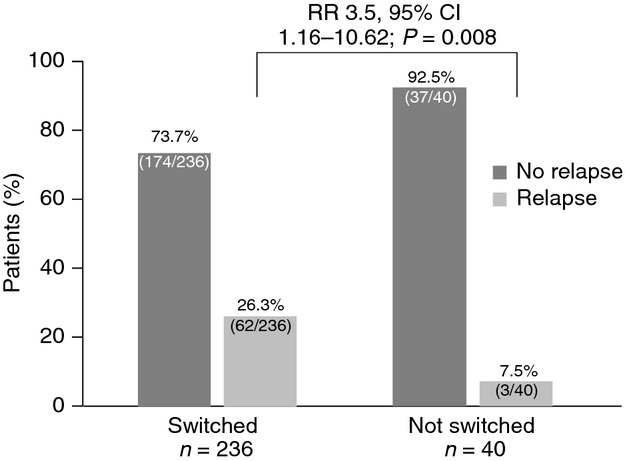
Risk of relapse in adherent patients who did and did not switch mesalazine formulation (adherent patients subgroup, *n* = 276). RR, risk ratio.

A further analysis of the effect of switch was conducted in the subgroup of patients who were adherent in the baseline period (MPR ≥80%; *n* = 276). The mean duration of follow-up in this analysis was 610.38 days (1.67 years; 95% CI 582.2–638.6 days). In these adherent patients, there was a significant relationship between switch and relapse (Figure 5). A total of 236/276 (85.5%) adherent patients were switched to an alternative mesalazine formulation. Of the adherent patients who switched, 26.3% (62/236) suffered relapses, compared to 7.5% (3/40) of those who did not switch (*P* = 0.010). This finding was confirmed after controlling for age, sex and adherence in the follow-up period (*P* = 0.012). These analyses indicate that switching is associated with a significant increase in the risk of relapse in adherent patients (RR 3.5, 95% CI 1.16–10.62; *P* = 0.008).

## Discussion

This study examined the impact of key factors that are postulated to destabilise maintenance therapy in IBD, focusing on non-adherence to long-term mesalazine therapy and switches between mesalazine formulations. The value of long-term maintenance therapy to prevent relapses in patients with IBD is well established,^1^–^2^ so understanding these factors represents an important priority.

The findings confirm that non-adherence is a widespread problem in the UK, with only 40% of patients classed as adherent. Indeed, this figure is lower than that observed in many previous studies; Jackson *et al*. reported that most adherence studies in IBD patients recorded adherence rates of 55–70%.^13^ Moreover, the significant rise in the risk of relapse associated with non-adherence highlights the clinical importance of improving adherence to support stable symptom control.

Interestingly, no significant differences in adherence were observed among formulations. The currently available formulations of mesalazine have a number of practical differences, including the administration regimens for which they are indicated. While certain regimens may suit some patients, altering administration regimens may not provide a universal solution to non-adherence. This suggests that more in-depth and sophisticated strategies will be required to improve adherence across the board.

Examining the impact of medication switches provides an important insight into an increasingly relevant issue. Many patients experience mesalazine switches at some point during their disease – many of these switches are for clinical reasons, yet others result from ambiguous or nonbranded prescriptions or from attempts to reduce prescribing costs. In particular, a number of regional health authorities in the UK have conducted programmes to switch patients between 5–ASA formulations – for example, in Dumfries and Galloway^14^ and in Surrey.^15^ Such programmes have generated controversy among gastroenterologists,^9^ so the clinical impact of mesalazine switches is an important area to research.

In the initial analysis of the full subgroup, there was no significant decrease in adherence or increase in relapses associated with medication switches. This is perhaps to be expected – in a population where non-adherence is prevalent, the impact of a change in formulation or mesalazine release profile may well be masked by non-adherence. Conversely, in those patients who were adherent, the impact of switch was striking. Adherent patients who switched to another mesalazine had a 3.5 times greater risk of relapse compared with nonswitched patients. Interestingly, the significant relationship was retained when controlling for age, sex and adherence after the switch. Thus, while some changes in mesalazine formulation are important and clinically justified, the evidence in this study suggests that when patients are stable on an existing formulation, they should not be switched unless clinically necessary.

Although this analysis was conducted on a single formulation of mesalazine (specifically the most commonly prescribed formulation, Asacol, to minimise confounding while ensuring sufficient statistical power), it would be interesting to establish whether this effect is observed with other formulations. Such a study represents a promising opportunity for further research.

Patient databases offer a potentially valuable source of observational data on clinical practice, although many remain underused.^16^ This is, in part, due to limitations in the data they contain and the technical and methodological challenges such studies pose. While clinical databases provide a direct record of clinical events, these data may be restricted by how often patients report relapses and how the relapses are coded when they do, making the data potentially unreliable. Conversely, pharmacy databases provide a broad picture of the medication that patients receive, and are comparatively unrestricted by the mixture of primary and secondary care involvement in IBD. In this way, pharmacy databases offer a valuable insight into patient behaviours through measures of adherence (although they do not explore the origins of these behaviours).

However, such databases do not record clinical events such as relapses, necessitating the use of a proxy measure. The current proxy measure is not validated, and it is not anticipated that it would necessarily identify all relapses, as other treatment approaches are available. For example, many relapses will be treated with steroids; in fact, steroid treatment has been used as a marker of relapse.^7^ For the present study, however, steroid prescription was not an appropriate measure for relapse, as most short courses of steroids are dispensed in hospital, and hence would not be adequately captured by the current methodology.

Doubling mesalazine dose is another of the possible therapeutic strategies for the treatment of relapses of active UC, and can be examined using a dispensing database. For example, if patients suffer a moderately active relapse while taking 2.4 g/day maintenance therapy, their doctor may elect to double the mesalazine dose to 4.8 g/day to treat the relapse, and such an approach would be broadly consistent with treatment guidelines from the BSG.^2^ As a result, the proxy measure used in this study is anticipated to identify a relevant subset of relapses. Moreover, even if some patients were not experiencing formally defined relapses, a doubling of mesalazine dose is a sign that there has been some form of destabilisation of disease control or suboptimal management that is sufficient to warrant clinical concern. As this proxy has not been validated, the findings must be interpreted with caution. However, in the absence of more robust real-world data (and, indeed, further research to this end would be highly valuable), the proxy allows us to exploit the currently available data resources to obtain a timely insight into this relevant clinical issue.

Further limitations of the results of this study arise from certain elements of the methodology and available data. Inclusion of only treatment-naïve patients was necessary to obtain a homogenous population and reduce confounding by patients' previous experiences, but limits the generalisability of the results. Furthermore, due to a high prevalence of missing and incomplete data in the database, a large number of patients were excluded; this represents a further limitation in the data, although it is noted that the sample sizes remain high. The database does not include details such as disease extent or severity or the reason why the switches were conducted, and such characteristics would be interesting to explore further.

In addition, it would be interesting to examine the timing of relapses relative to a switch, and their durations – the current methodology allows the occurrence of a relapse to be identified, but does not allow assessment of timing. Although some such information could be gleaned from the proxy, it would be limited in precision to the gap between prescriptions – in many cases, as much as 3–6 months. That said, if a switch were simultaneously accompanied by a doubling of dose, this would not have been recorded as a relapse in the follow-up period; consequently, all flares recorded in the follow-up period were experienced after the switch, and not concurrently. Nevertheless, it is not possible to infer a causal link between switches and relapses based on the current evidence; indeed, even with detailed timing information, the unpredictable and multi-factorial nature of relapses and the variations among patients in when they present for treatment would make a temporal link difficult to establish. The strong association observed in this study is sufficient to advocate caution when considering switching stable patients.

This study did not specifically look at the dosage of mesalazine that patients were taking. This may therefore represent a confounding factor, as well as another opportunity for further research. For example, if switches were accompanied by a change in dose, it may be the change in dose that disrupts disease control, rather than the switch itself. However, as switches accompanied by a doubling of dose were not recorded as relapses, the observed rates of relapse are not influenced by clinicians electing to treat a relapse by switching mesalazine treatment to another formulation at a doubled dose. More importantly, even if dosage were acting as a confounding factor in this way, the implication for clinical practice remains the same: altering the treatment of a patient who is otherwise stable is associated with a rise in the risk of relapse and hence should be avoided.

The limitations of this study imply that the results should be interpreted with caution, and clearly show that further research would be valuable. Nevertheless, the findings indicate that non-adherence and mesalazine switches are potentially serious issues for disease control that warrant further consideration. The current methodology provides a practical and pragmatic approach to obtain evidence based on real-world patients. By controlling for age and sex and focusing on one mesalazine formulation in isolation, the confounding effects of these variables are minimised, while ensuring that the data remain applicable to real clinical settings.

The need for further research into the potential links among mesalazine adherence, switches and relapses of active IBD is pressing, to ensure that patients are not exposed to an unnecessary risk of relapse. At the same time, this could have considerable economic implications; if switch programmes are conducted to reduce mesalazine prescribing costs, an increased risk of relapse could have a dramatic effect on their overall cost effectiveness. In the meantime, the findings of the current study provide sufficient evidence to advocate caution when considering mesalazine switches for stable patients.

## Authorship

*Guarantor of the article*: Gwen Wiseman.

*Author contributions*: AR was involved in study conception and design and interpretation of the data. MH was involved in the design and conduct of the database analyses and statistical evaluations. GW was involved in the study conception, design and interpretation. MJ designed and performed the database analyses. All authors contributed to the development of the manuscript, and all approved the final version.
